# Leukoencephalopathy with calcifications and cysts: A case report

**DOI:** 10.1097/MD.0000000000007597

**Published:** 2017-07-21

**Authors:** Yubao Ma, Xingwen Zhang, Chen Cheng, Quangang Xu, Hai Di, Jiao Zhao, Dehui Huang, Shengyuan Yu

**Affiliations:** a Department of Neurology, Chinese PLA General Hospital, Beijing; b Department of Neurology, Tianjin Medical University General Hospital, Tianjin, People's Republic of China.

**Keywords:** calcification, cyst, genetic mutation, Labrune syndrome, leukoencephalopathy

## Abstract

**Rationale:**

Leukoencephalopathy with calcifications and cysts (LCC) is an uncommon entity characterized by edematous leukoencephalopathy, cerebral calcifications, and parenchymal cysts. Due to its rarity, the clinical, radiological, and histopathological features have yet to be well elucidated.

**Patient concerns:**

The first case is a 35-year-old female who was asymptomatic. A giant intracranial cyst was incidentally detected radiologically, and it was slowly growing in the recent 10 years. The second case is a 20-year-old female who presented with a 1-month history of headache. Brain computed tomography showed multiple asymmetric calcifications in the bilateral basal ganglia and white matter. Magnetic resonance imaging revealed a cyst in the right parietal lobe.

**Diagnoses:**

They were diagnosed with LCC.

**Interventions and Outcomes:**

The first patient underwent surgical resection of the intracranial cyst, and the second patient received a stereotactic biopsy. The patients performed well postoperatively.

**Lessons:**

LCC can be found at any age. A young age seems to be associated with severer symptoms. The clinical manifestations can be variable and aggressive. The potential pathogenic basis still needs further research.

## 1 Introduction

Leukoencephalopathy with calcifications and cysts (LCC) is an uncommon entity that is radiologically characterized by edematous leukoencephalopathy, cerebral calcifications, and formation of parenchymal cysts.^[1–3]^ LCC was first reported by Labrune in 1996, and thus, it was also known as Labrune syndrome.^[4]^ The current literature regarding LCC is primarily limited to case reports and does not provide a comprehensive overview. Thus, at present, the clinical, radiological, and histopathological features have yet to be well elucidated.

Herein, we report 2 another cases of LCC. In addition, we reviewed the literature and identified a total of 25 relevant studies reporting on 31 patients with LCC. The individual clinical manifestations, radiological characteristics, and pathological findings were summarized and analyzed.

## 2 Case presentation

The study was approved by the Ethical Committee of the general hospital of Chinese People's Liberation Peking, China. Written consent was obtained.

### 2.1 Case 1

**Figure 1:**
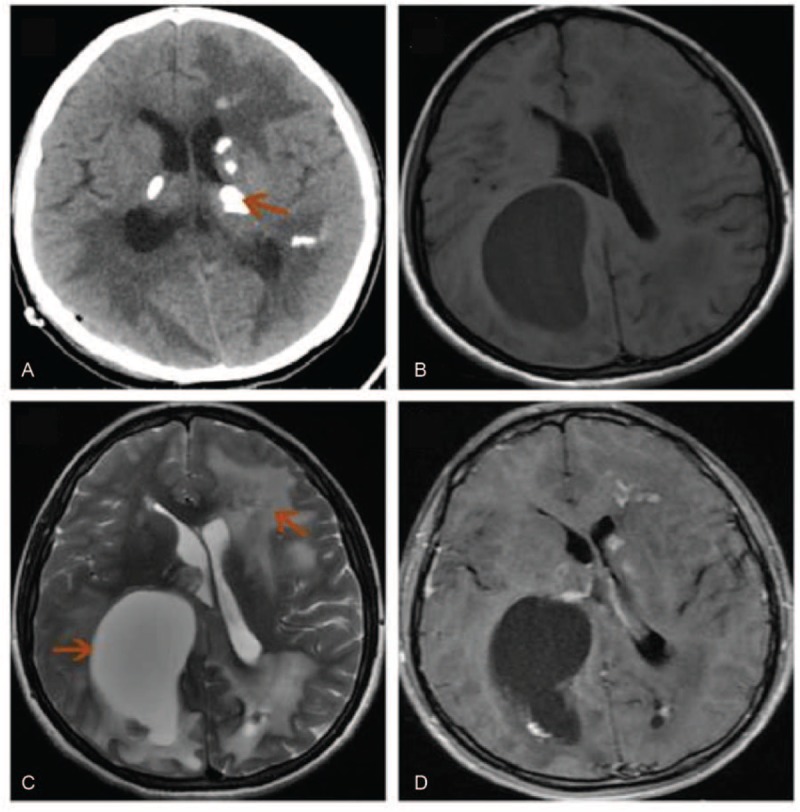
Radiological profiles of Case 1. (A) Brain computed tomography showed multiple asymmetric calcifications in the bilateral basal ganglia. (B) Magnetic resonance T1-weighted imaging showed a giant cyst in the right parietal lobe and hypointensities in the bilateral white matter. (C) Magnetic resonance T2-weighted imaging showed a giant cyst in the right parietal-occipital lobe and extensive hyperintensities in the bilateral white matter. (D) Magnetic resonance contrasted T1-weighted imaging showed nodular enhancement along the cyst wall and focal enhancement in the white matter.

**Figure 2:**
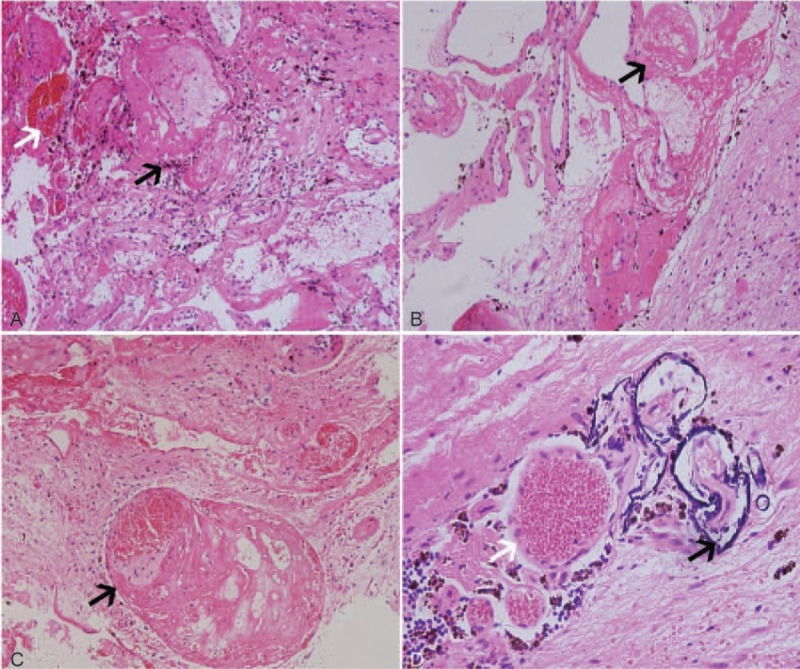
Pathological images of Case 1. (A, × 20) Pathological examination showed angiomatoid hyperplasia with cellulose-like degeneration, in the vascular wall and vascular congestion (white arrow), and intervascular hemosiderosis was visible (black arrow). (B, × 20) The angiomatoid hyperplasia was well defined, and the black arrow indicates vascular fibrinoid exudation. (C, × 20) Solitary vascular stenosis with cellulose-like exudation and recanalization (black arrow) was visible. (D, × 40) Circular iron deposition with surrounding hemosiderin (black arrow) and vascular congestion (white arrow) was visible.

The patient was a 35-year-old woman in whom an intracranial cystic lesion in the right temporal-parietal lobe and multiple calcifications in the bilateral basal ganglia were incidentally detected by computed tomography (CT) and magnetic resonance imaging (MRI) 10 years previously. There was no significant symptom or sign. A biopsy was performed, but the histopathology did not reveal any abnormality. In the following years, repeated MRIs showed the lesion was slowly growing, and the patient remained asymptomatic. On this admission, the brain MRI showed that the lesion was significantly enlarged compared with its size at the initial radiological examination (Fig. 1). The patient denied any family history. The examination of ocular fundus was normal. The antibody tests for syphilis and human immunodeficiency virus (HIV) were both negative. A cystectomy was performed; and intraoperatively, the lesion was pink, hard, and well-demarcated with a rich blood supply and yellowish-brown cystic component. Pathological examination showed vascular tumor-like hyperplasia with congestion and hemorrhage, suggesting a vasogenic lesion (Fig. 2). A diagnosis of LCC was made.

### 2.2 Case 2

**Figure 3:**
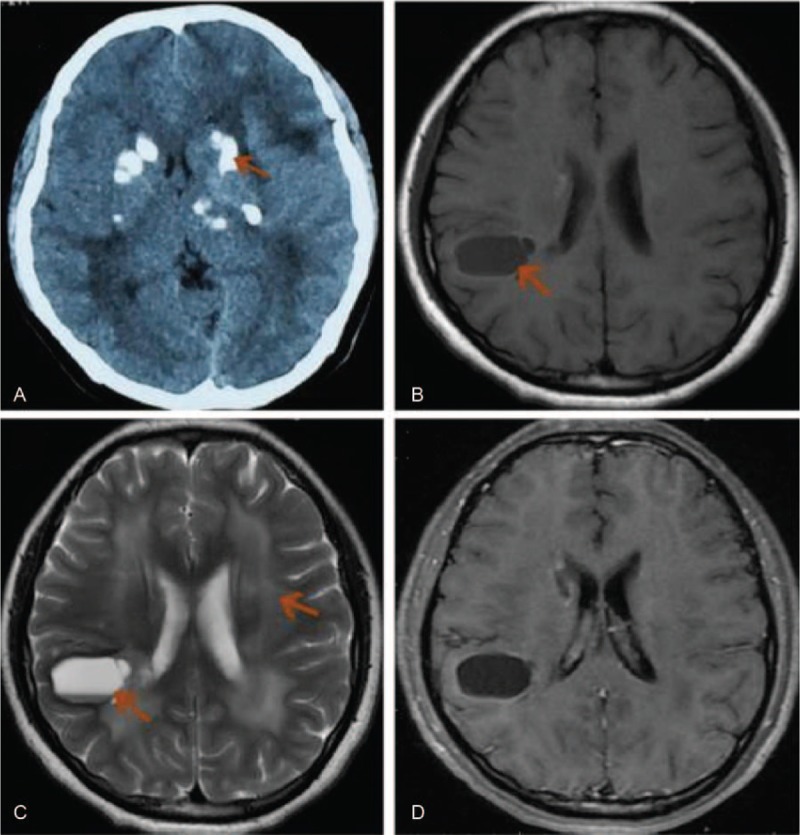
Radiological profiles of Case 2. (A) Brain computed tomography showed multiple asymmetric calcifications in the bilateral basal ganglia and white matter. (B) Magnetic resonance T1-weighted imaging showed a cyst in the right parietal lobe with deposit of hemosiderin. (C) Magnetic resonance T2-weighted imaging showed a giant cyst in the right parietal lobe and hyperintensities in the bilateral white matter. (D) Magnetic resonance contrasted T1-weighted imaging showed ring-like enhancement along the cyst wall.

**Figure 4:**
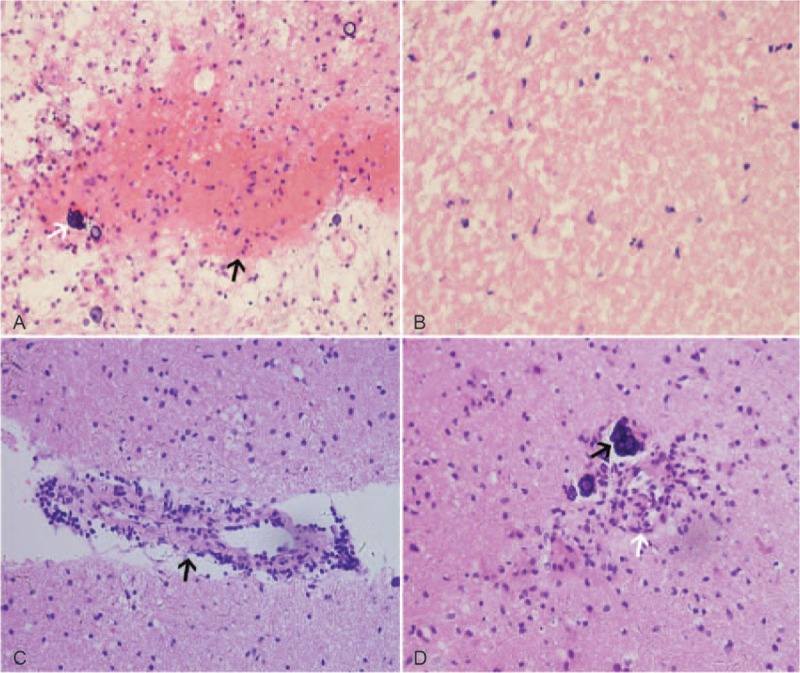
Pathological images of Case 2. (A, × 20) Pathological examination showed hemorrhage (black arrow) with surrounding loose tissue and calcification (white arrow). Necrosis (B, × 40) and sleeve-like lymphocyte accumulation along vessels (C, × 40) were visible. (D, × 40) Perivascular calcification (black arrow) and intravascular neutrophils (white arrow) were visible.

A 20-year-old female presented with a 1-week history of tension-type and progressive headache. Three days before admission, the headache became aggravated, and she developed nonprojectile vomiting. Mini Mental State Examination (MMSE) revealed a total score of 29 points. Physical examination showed tendon reflex hyperfunction, and the patient was positive for bilateral Hoffman signs, Chadock signs, and orbicularis oris reflexes. The muscle strength was normal (grade 5/5). The patient denied any family history. The examination of ocular fundus was normal. Cranial CT demonstrated an oval lesion with heterogeneous density and extensive perilesional edema, and the midline was shifted. Cystic lesions were noted in the right hemisphere, and asymmetrical calcifications were observed in the bilateral basal ganglia. On brain MRI, the oval lesion in the left frontal-temporal lobe appeared heterogeneously hypointense on T1-weighted imaging, heterogeneously hyperintense on T2-weighted imaging, and hyperintense on diffusion-weighted imaging. After administration of contrast medium, ring-like and heterogeneous enhancement was noted. In addition, cystic lesions were visible in the right hemisphere (Fig. 3). On lumbar puncture examination, the routine, biochemical, immunological, and microbial parameters of cerebrospinal fluid were all normal. Mannitol was administered to lower the intracranial hypertension, and the symptoms were relieved immediately. A stereotactic biopsy was performed. Pathological examination showed leukoaraiosis, hemorrhage, calcifications, and lymphocyte accumulation, and immunohistochemical staining excluded tumor and infection (Fig. 4). Genetic analysis showed no abnormality in the *CTC1* gene. Based on the typical radiological and histological characteristics, a diagnosis of LCC was made.

## 3 Discussion

In the published literature, we identified a total of 31 cases of LCC, and the clinical, radiological, and pathological profiles of these cases are summarized in Table 1.^[1,2,4–26]^

**Table 1:**
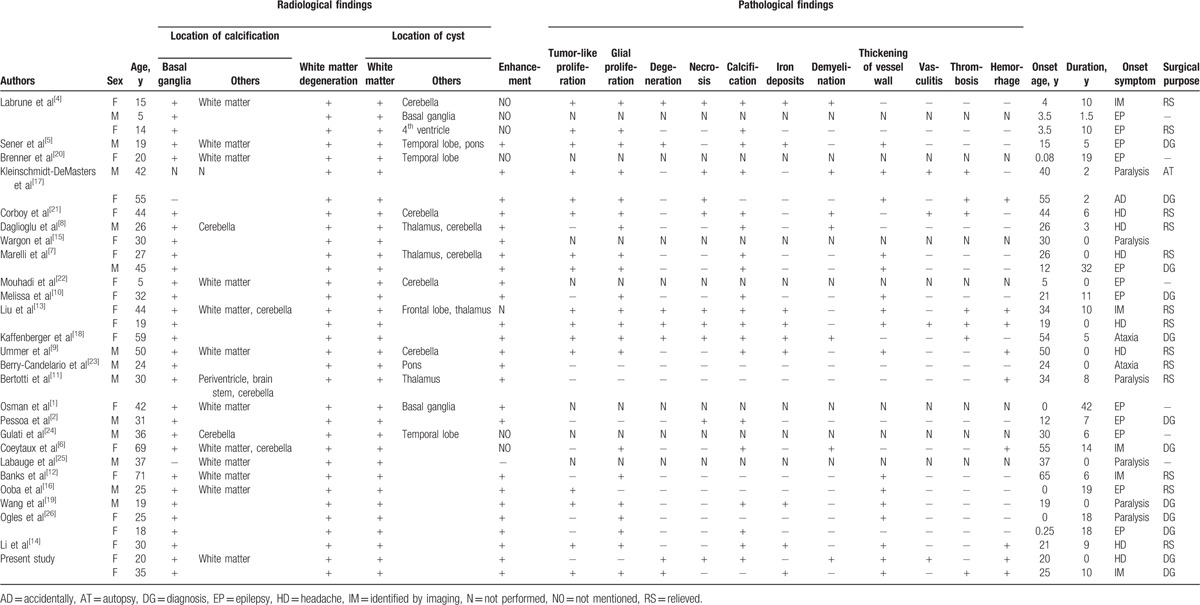
Clinical, radiological, and pathological features of LCC cases.

Including the current 2 cases, the 33 cases reviewed included 13 males (39.4%) and 20 females (60.6%), with a male-to-female ratio of 2:3. The onset age ranged from 5 to 71 years, with a median of 30 years (mean, 32.2 years, SD = 16.3 years).

The clinical manifestations included paralysis (n = 19, 57.6%), headache (n = 16, 48.5%), epileptic seizure (n = 12, 36.4%), ataxia (n = 11, 33.3%), cognitive dysfunction (n = 9, 27.3%), papilledema (n = 5, 15.2%), dystonia (n = 3, 9.1%), and visual disturbance (n = 3, 9.1%). Furthermore, 5 cases were asymptomatic. Nine cases were diagnosed within 1 year from onset, and the duration from onset to diagnosis in the other 24 cases ranged from 1.5 to 42 years (mean, 11.4 years).

Radiologically, CT data were available in 32 cases. Calcifications were located in the degenerative cerebral parenchyma. Calcifications in the basal ganglia were noted in 30 (93.8%) cases, among which concomitant white matter calcifications were present in 13 (40.6%) cases and cerebellar calcifications in 4 (12.5%) cases; 1 case only showed white matter calcifications without basal ganglia calcification; and calcification was absent in 1 case. MRI data were accessible for all 33 cases, and they all showed white matter degeneration and cystic lesions. Cystic lesions were primarily located in the bilateral centrum semiovale: 6 (18.2%) cases had cerebellar cysts, 3 (9.1%) cases had pons cysts, 6 (18.2%) cases had basal ganglia cysts, and 4 (12.1%) cases had hemorrhagic cysts. The involved brain regions were nonspecific and variable, including the supratentorial sites, infratentorial sites, cortex, white matter, cerebella, and brain stem. Contrast MRI was performed in 27 patients, and significant enhancement appearing mostly as ring-like enhancement and seldom as focal enhancement was observed in 25 (92.6%) cases. Only 1 case showed no enhancement.

The treatments included medications and surgery. Till now, there has been no medication with specific efficacy for LCC, and the current widely used medications are antiepileptic drugs (AEDs) and dehydrant agents. Some patients with progressive intracranial hypertension because of significant progression of cysts necessitate surgical decompression. In the current study, 13 (39.4%) cases underwent surgical treatment to relieve intracranial hypertension.

Pathological examination was performed in 26 (78.8%) cases. Vascular tumor-like hyperplasia was observed in 16 (61.5%) cases, Renshaw cell and glial proliferation in 21 (80.8%) cases, calcification in 19 (73.1%) cases, hyperplasia of vessel wall in 16 (61.5%) cases, necrosis in 10 (38.5%) cases, degeneration in 7 (26.9%) cases, iron deposition in 9 (34.6%) cases, demyelination in 7 (26.9%) cases, vascular inflammatory changes in 4 (15.4%) cases, thrombosis in 7 (26.9%) cases, and hemorrhage in 8 (30.8%) cases. Only 1 (3.8%) case showed no specific change.

Five (15%) patients from 2 families were identified as having familial LCC; nevertheless, no genetic analysis was performed. In the current study, we performed a genetic screening on the *CTC1* gene, and the result was negative.

Cases of LCC have been reported all over the world, with no significant ethnic predominance. LCC can be found in any age, with the majority of cases being reported in young and middle-aged patients, with a slight female predominance.

The brain involvement in LCC can be extensive. The clinical manifestations are nonspecific, including paralysis, headache, epileptic seizure, ataxia, cognitive dysfunction, papilledema, dystonia, and visual disturbance. Approximately 66.7% of patients with cognitive dysfunction were younger than 12-years old, indicating that LCC may retard mental development.^[5,6]^ The long duration from onset to diagnosis suggests a slow progression of LCC.^[7]^ Intracranial hypertension was the main cause for emergency in LCC, necessitating surgical treatment.^[8]^ Preoperative diagnosis of LCC based only on clinical and radiological profiles is quite challenging, and definitive diagnosis depends on pathological biopsy.^[6,9]^

The typical characteristics on neuroimaging include extensive white matter degeneration and asymmetric cysts and calcifications. On contrast MRI, the lesions appear as ring-like enhancement along the cyst wall or nodular enhancement in the degenerated white matter, indicating that LCC is associated with angiogenesis.^[10,11]^ The leukoaraiosis and formation of calcifications suggest the presence of tissue degeneration and necrosis.^[11,12]^ The spectrum analysis conducted by Sener et al^[5]^ found the main component of cystic fluid was lactate, indicating the formation of cysts may be associated with tissue ischemia and hypoxia. In addition, bleeding deposits could be found in the cysts in a few cases, also suggesting the formation of cyst is related to hemorrhage.^[13,14]^ The radiological and pathological findings both support that LCC is associated with the involvement of small vessels.^[14,15]^ In our second case, intracystic hemorrhage was noted on MRI, and we speculated that this might be the cause of headache aggravation. The occurrence of intracranial hypertension mostly depends on the location of cysts. From the literature review, we found that patients with cerebellar or pons cysts were more likely to develop intracranial hypertension, which may be the result of space-occupying effects and aqueductal stenosis.^[16]^

Pathologically, LCC primarily manifests as vascular tumor-like hyperplasia, Renshaw cell and glial proliferation, calcification, degeneration, necrosis, and hemorrhage. The calcifications and hemosiderosis along the vessel walls suggest necrosis and hemorrhage of small vessels. About 22% of reported cases showed thickening and degeneration of vessel walls and lymphocyte aggregation, further supporting the pathological change of small vessels.^[4,5,9,15,17,18]^ We speculated that the main cause of tissue degeneration, calcification, and necrosis in LCC might be tissue ischemia and hypoxia as a result of repeated vasculopathy.

The differential diagnosis should include Coat plus syndrome, which shares similar radiological presentations with LCC. Coat plus syndrome is reported with obvious inherited tendency and earlier onset age, and patients usually present with typical retinal angioma; genetic analysis revealed a mutation in the *CTC1* gene. In the literature review, only 5 cases were found to have an inherited tendency. We hypothesize that LCC and Coat plus syndrome may share the similar genetic basis^[19]^; nevertheless, no *CTC1* mutation was identified in the current study. The definitive pathogenetic variants may be a hot topic in future studies.

## 4 Conclusions

LCC is a rare disorder radiologically characterized by extensive white matter degeneration, asymmetric cerebral calcifications, and formation of parenchymal cysts. The definitive pathogenetic mechanism still needs further research.
